# Volume-controlled ventilation versus pressure-controlled ventilation during spine surgery in the prone position: A meta-analysis

**DOI:** 10.1016/j.amsu.2022.103878

**Published:** 2022-05-25

**Authors:** Jun Han, Yunxiang Hu, Sanmao Liu, Zhenxin Hu, Wenzhong Liu, Hong Wang

**Affiliations:** a Department of Spine Surgery, Dalian Municipal Central Hospital Affiliated of Dalian Medical University, Dalian, 116033, Liaoning, China; b Department of Spine Surgery, The First Affiliated Hospital of Dalian Medical University, Dalian, 116011, Liaoning, China; c Dalian Medical University, Dalian, 116044, Liaoning, China; d Department of Joint Surgery, Gaomi People's Hospital, Gaomi, 261500, Shandong, China

**Keywords:** Volume-controlled ventilation, Pressure-controlled ventilation, Spine surgery, Prone position, Randomized controlled trials, Meta-analysis, PILF, Posterior Lumbar Interbody Fusion, RCT, randomized controlled trial, NP, not provided, PCV, pressure controlled ventilation, VCV, volume controlled ventilation, ASA, American Society of Anesthesiologists, VT, tidal volume, PEEP, Positive End Expiratory Pressure, BMI, body mass index, MD, mean difference, IOB, intra-operation blood loss, POB, post-operation blood loss, Hb, hemoglobin, HCT, hematocrit, CVP, central venous pressure, HR, heart rates, MAP, mean arterial pressure, Cdyn, dynamic compliance

## Abstract

**Background:**

Many studies have investigated a comparison of the potency and safety of PCV versus VCV modes in spinal surgery in prone position. However, controversy about the maximal benefits of which ventilation modes remains. The main purpose of this meta-analysis was to investigate which one is the optimal ventilation for surgery patients undergoing spine surgery in prone position between the two ventilation modes as PCV and VCV.

**Methods:**

We conducted a comprehensive search of PubMed, Embase, Web of Science, the Cochrane Library, and Google Scholar for potentially eligible articles. The continuous outcomes were analyzed using the mean difference and the associated 95% confidence interval. Meta-analysis was performed using Review Manager 5.4 software.

**Results:**

Our meta-analysis included 8 RCTs involving a total of 454 patients between 2012 and 2020. The results demonstrated that IOB, Ppeak and CVP for VCV are significantly superior to PCV in spinal surgery in prone position. And PCV had higher Cdyn and PaO_2_/FiO_2_ than VCV. But there was no significant difference between PCV and VCV in terms of POB, Hb, HCT, HR and MAP.

**Conclusions:**

The PCV mode displayed a more satisfying effect than VCV mode. Compared to VCV mode in same preset of tidal volume, the patients with PCV mode in prone position demonstrated less IOB, lower Ppeak and CVP, and higher PaO_2_/FiO_2_ in spinal surgery. However, there is no obvious difference between PCV and VCV in terms of hemodynamics variables (HR and MAP).

## Highlights

• The PCV mode displayed a more satisfying effect than VCV mode.
• Compared to VCV mode, the patients with PCV mode in prone position demonstrated less IOB, lower Ppeak and CVP, and higher PaO2/FiO2 in spinal surgery.
• There is no obvious difference between PCV and VCV in terms of hemodynamics variables.

## 1 Introduction

Mechanical ventilation is broadly required for patients who undergo a wide variety of surgeries under general anesthesia but may cause alveolar overstretching and ventilation-associated heart–lung injury while maintaining the stability of their cardiopulmonary function [1,2]. Apart from the ventilation method applied, use of the prone position for patients undergoing spinal surgery may lead to some changes in cardiopulmonary function. This scenario may cause inferior vena cava obstruction and increased thoracic pressure, which lead to a decreased cardiac index [3,4]. Thus, the common coexistence of mechanical ventilation and prone position could exert a cumulative effect on cardiopulmonary function, further affecting the safety of the surgical process and the patient's prognosis [5].

Among the many mechanical ventilation modes, VCV and PCV are the two most commonly used modes during prone position spinal surgery. VCV implements ventilation with a preset ventilation volume, and tidal volume, positive end-expiratory pressure (PEEP). Respiratory rate and expiration/inspiration ratio (E/I ratio) are controlled by the anesthetist. In contrast, the parameters controlled by the anesthetist are the peak and plateau inspiration pressures and the E/I ratio. Additionally, attention should be given to pulmonary compliance and airway resistance to monitor the airway pressure in PCV and the tidal volume in VCV.

In recent years, a number of studies have analyzed the effects of different ventilation patterns on the hemodynamics of patients undergoing spinal surgery during the entire process, but no unified conclusion has been reached regarding the use of either VCV or PCV mode [6,7]. During prone position, we hypothesized that VCV and PCV would have different hemodynamic effects to patients undergoing spinal surgery; Therefore, for patients undergoing spine surgery in prone position, it is worth exploring which ventilation mode is optimal. Based on our knowledge, no relevant published meta-analysis has investigated the efficacy and safety of PCV versus VCV in spine surgery with prone position. We performed this meta-analysis on multiple RCTs to compare the efficacy and safety of PCV versus VCV during spine surgery for patients in prone position.

## 2 Materials and method

This Meta-analysis was carried out following Preferred Reporting Items for Systematic Reviews and Meta-Analyses (PRISMA) guidelines [8]. The assessment of multiple systematic reviews (AMSTAR) 2 tool were used to evaluate quality of included articles [9]. The protocol for this meta-analysis is available in PROSPERO (CDR42020196916). Efficacy and safety were categorized into the primary outcome and secondary outcome. Specifically, our primary outcome was the amount of intraoperative blood loss (IOB), while secondary outcomes were postoperative blood loss (POB), peak inspiratory pressure (Ppeak), hemoglobin (Hb), hematocrit (HCT), central venous pressure (CVP), heart rate (HR), mean arterial pressure (MAP), dynamic compliance (Cdyn), and PaO_2_/FiO_2_.

### 2.1 Search strategy

To retrieve all potentially eligible studies, two researchers (***) independently screened multiple databases, including PubMed, Web of Science, Embase, the Cochrane Library as well as Google Scholar. The following keywords combined with free words were used: “Pressure-controlled”, “Pressure-controll”, “Pressure controlled”, “Volume-controlled”, “Volume -controll”, “Volume controlled”, “Ventilation”, “Ventilator”, “Prone position”, “Surgery”, “Surgical”, “Operation” with the Boolean operators “AND or OR”. There was no language restriction, and the last search was updated in June 2020. Additionally, all identified publications' reference lists and other meta-analysis were manually searched as well.

### 2.2 Inclusion and exclusion criteria

The following criteria were used for inclusion and exclusion: (1) population: all patients were adults and underwent spine surgery in prone position; (2) study design: RCT; (3) interventions: VCV and PCV; and (4) the given study included at least one of the following outcomes: IOB, POB, Ppeak, Hb, HCT, CVP, HR, MAP, Cdyn, and PaO_2_/FiO_2_. The following criteria were used as exclusion criteria: (1) animal studies; (2) other surgery; (3) serious liver or kidney disorder or a respiratory or circulatory disease; and (4) case reports, comments, reviews, letters as well as editorials.

### 2.3 Data extraction and quality assessment

Data from all enrolled studies were extracted by two independent observers (***), and in the event of a discrepancy, a third author (***) was consulted for consensus. The general features included the first author, publication year, country, study type, number of enrolled participants (PCV:VCV), surgical approach, age, gender, body mass index (BMI), anesthesia approach, and ventilatory intervention (PCV:VCV).

Two observers (***) performed a quality assessment of all RCTs following the Cochrane Handbook for Systematic Reviews [10]. The evaluation of bias consisted of 7 sections that included the following elements: (1) random sequence generation; (2) concealment of allocation; (3) blinding of participants and personnel; (4) blinding of the outcome assessment; (5) incomplete outcome data; (6) selective reporting; and (7) other bias. Based on the actual study content, each section had a high, low, or unclear bias risk.

### 2.4 Statistical analysis

The meta-analysis of comparable data was performed using Review Manager 5.4 software. Continuous outcomes, such as IOB, POB, Ppeak, Hb, HCT, CVP, HR, MAP, Cdyn, and PaO_2_/FiO_2_, were analyzed using the mean difference (MD) and the 95% CI. χ^2^ and *I*_*2*_ tests were used to assess data heterogeneity. We conducted a heterogeneity test on all included studies and calculated the inconsistency index (*I*_*2*_) statistic. When *I*_*2*_>50% or P value < 0.1, significant heterogeneity of the recruited studies was indicated, and a random-effect model was adopted. Otherwise, a fixed-effect model was adopted. The z test was used to determine the pooled effects, and a P value < 0.05 was considered statistically significant. For several comparisons, one-way sensitivity analysis were conducted by removing publications individually to assess the robustness of the results.

Notably, in the studies conducted by Li et al. [11] and Kang et al. [6], the parameters Ppeak, HR, MAP, and CVP were measured at multiple time points during operations in prone position. In these cases, we chose two time points in these studies and merged their data as representative for the study. In addition, for data displayed as medians (25–75%, interquartile range), We converted the median (interquartile range) into the mean (standard deviation) by using a conversion formula that is commonly accepted in the literature [12].

## 3 Results

### 3.1 Study selection

**Fig. 1:**
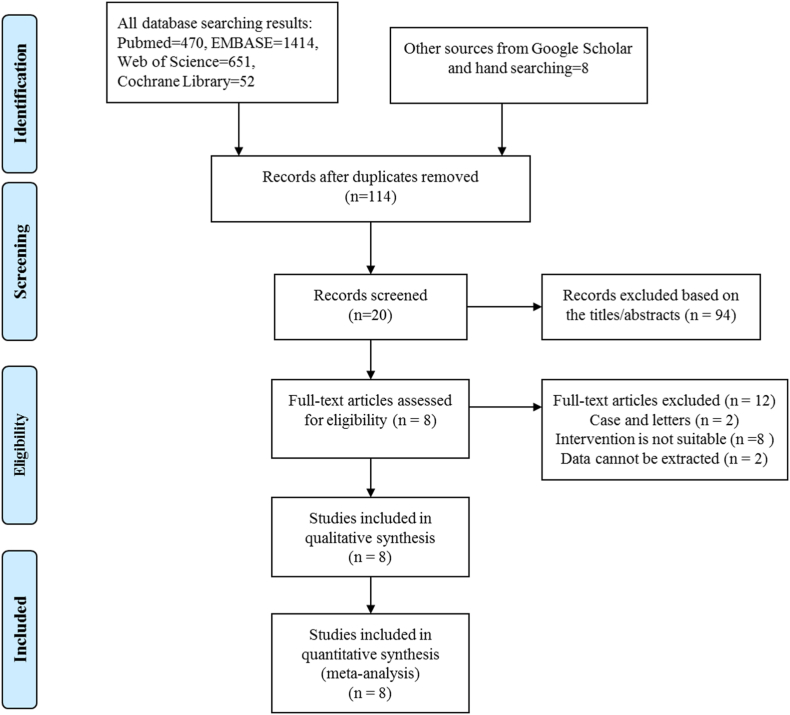
Flow diagram of the study selection process for the meta-analysis.

A total of 2587 relevant studies were enrolled according to the searching strategy, and 8 RCTs [6,11,[13], [14], [15], [16], [17], [18]] were ultimately selected for meta-analysis. Fig. 1 shows the flowchart of the literature exclusion and inclusion stages, the reasons and number of excluded studies, and finally the number of articles.

### 3.2 Characteristics of selected studies

In all, 8 RCTs studies involving a total of 454 patients were enrolled in our meta-analysis between 2012 and 2020. Table 1 summarizes the basic characteristics of the 8 enrolled studies. Two studies were conducted in Korea, 4 studies were performed in China, 1 study was conducted in Egypt, and 1 study was conducted in Turkey. Three studies reported specific surgical methods, such as posterior lumbar interbody fusion (PLIF); however, the other 5 studies only provided ambiguous surgical methods: 3 lumbar surgeries and 2 spinal surgeries. Two studies [6,11] involved a comparison of postoperative blood loss in PCV versus VCV in patients undergoing prone position spinal surgery; Li et al. calculated POB over a period of 96 h, while Kang et al. used a 72 h period. Only 5 definitive diseases were reported in two [11,13] of all the included articles in our meta-analysis; Zhou et al. described lumbar disc herniation, lumbar spondylolisthesis and lumbar fracture, and Li et al. described lumbar tuberculosis and lumbar tumors.

**Table 1:** Characteristics of all the trials included in the meta-analysis.

Study	Country	Study type	Surgical methods	Disease diagnosis	ASA (range)	Anesthesia methods	Mechanical ventilation	VT (ml/kg)	FiO2(%)	PEEP (cmH2O)	Age (years)	Gender M: F	BMI (kg/m2)
Şenay 2016	Turkey	RCT	spinal surgery	NP	**I-III**	General anesthesia	PCV VCV	6-8 ^**†**^ 6–8^**‡**^	NP ^**†**^ NP^**‡**^	20 ^**†**^ 20^**‡**^	64.0 ± 7.3 ^**†**^ 65.9 ± 7.5^**‡**^	18/3 ^**†**^ 10/10^**‡**^	P ^**†**^ NP^**‡**^
Amir 2020	Egypt	RCT	PILF	NP	**I-II**	General anesthesia	PCV VCV	8-10 ^**†**^ 8–10^**‡**^	100%^**†**^ 100%^**‡**^	5^**†**^ 5^**‡**^	44.4 ± 7^**†**^ 43.5 ± 5.4^**‡**^	21/25^**†**^ 19/27^**‡**^	27.8 ± 1.7^**†**^ 27.3 ± 1.9^**‡**^
Jo 2012	Korea	RCT	lumbar surgery	NP	**I-II**	General anesthesia	PCV VCV	10 ^**†**^ 10^**‡**^	50%^**†**^ 505^**‡**^	0^**†**^ 0^**‡**^	NP^**†**^ NP^**‡**^	NP^**†**^ NP^**‡**^	NP^**†**^ NP^**‡**^
Kang 2016	Korea	RCT	PILF	NP	NP	General anesthesia	PCV VCV	8 ^**†**^ 8^**‡**^	25%^**†**^ 25%^**‡**^	0^**†**^ 0^**‡**^	64 ± 13^**†**^ 66 ± 9^**‡**^	9/19^**†**^ 8/20^**‡**^	24.8 ± 3.3^**†**^ 26.0 ± 3.7^**‡**^
Bi 2014	China	RCT	spinal surgery	NP	**I-II**	General anesthesia	PCV VCV	10 ^**†**^ 10^**‡**^	50%^**†**^ 50%^**‡**^	0^**†**^ 0^**‡**^	49 ± 15^**†**^ 48 ± 14^**‡**^	10/10^**†**^ 11/9^**‡**^	25 ± 4^**†**^ 25 ± 4^**‡**^
Zhou 2013	China	RCT	lumbar surgery	lumbar disc herniation, lumbar spondylolisthesis, lumbar fracture	**I-II**	General anesthesia	PCV VCV	8 ^**†**^ 8^**‡**^	100%^**†**^ 100%^**‡**^	NP^**†**^ NP^**‡**^	53 ± 8^**†**^ 54 ± 7^**‡**^	17/13^**†**^ 16/14^**‡**^	NP^**†**^ NP^**‡**^
Peng 2017	China	RCT	PILF	NP	**I-II**	General anesthesia	PCV VCV	8 ^**†**^ 8^**‡**^	70%–80% ^**†**^ 70%–80%^**‡**^	NP^**†**^ NP^**‡**^	55.35 ± 9.57^**†**^ 59.85 ± 11.10^**‡**^	NP^**†**^ NP^**‡**^	NP^**†**^ NP^**‡**^
Li 2017	China	RCT	lumbar surgery	lumbar tuberculosis, lumbar tumor	**I-II**	General anesthesia	PCV VCV	8 ^**†**^ 8^**‡**^	25% ^**†**^ 25%^**‡**^	0^**†**^ 0^**‡**^	45.8 ± 11.9^**†**^ 47.2 ± 12.3^**‡**^	25/19^**†**^ 27/17^**‡**^	26.1 ± 4.2^**†**^ 25.3 ± 4.7^**‡**^

PILF, Posterior Lumbar Interbody Fusion; RCT, randomized controlled trial; NP, not provided; PCV, pressure controlled ventilation; VCV, volume controlled ventilation; ASA, American Society of Anesthesiologists; VT, tidal volume; PEEP, Positive End Expiratory Pressure; BMI, body mass index; ^**†**^, PCV; ^**‡**^, VCV.

### 3.3 Risk of bias

**Fig. 2:**
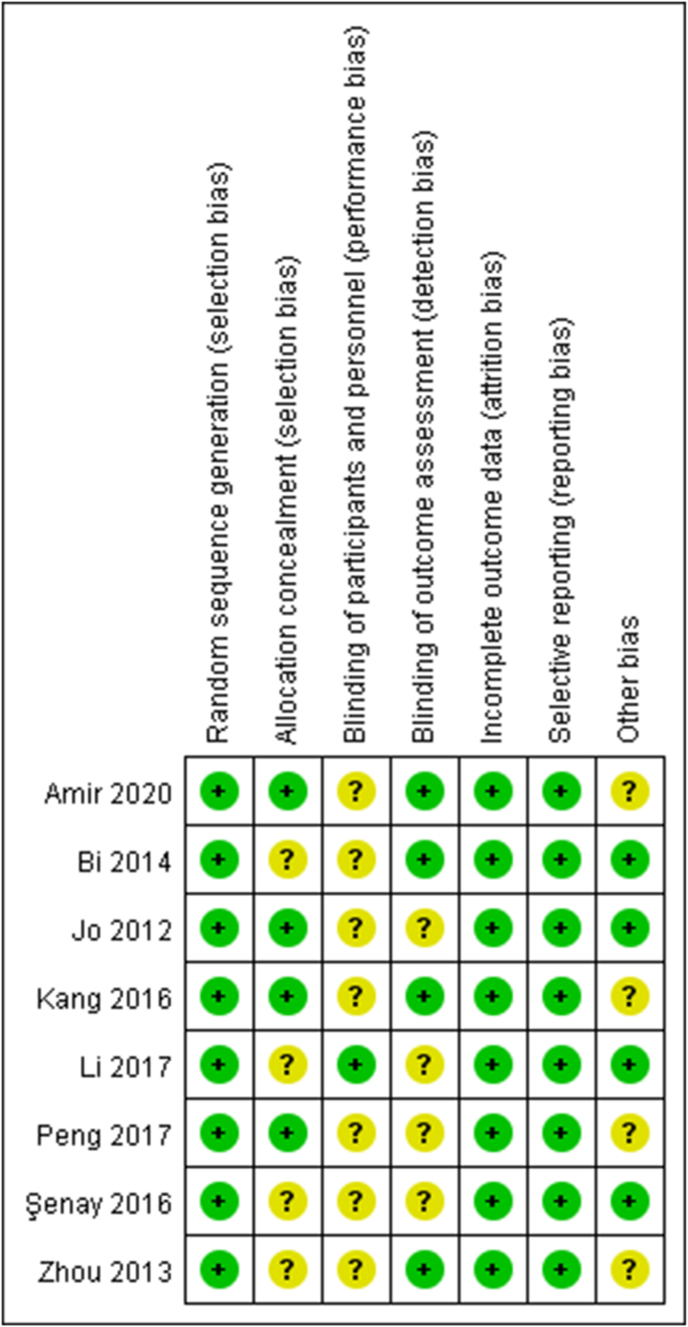
Risk of bias summary.

The risk of bias assessment results of the 8 RCTs are presented in Fig. 2. There was a low risk of bias in the 8 studies. Random sequence generation was observed in all 8 studies. Allocation concealment and blinding of the outcome assessment were noticed in 6 studies. Blinding of the participants and personnel were found in 1 study. The 8 studies did not present incomplete results data, selective reports, or other biases.

### 3.4 Results of the meta-analysis

The outcomes of the meta-analysis after a careful reading and analysis of the 8 RCT articles are presented in Table 2, including IOB, POB, Ppeak, Hb, HCT, CVP, HR, MAP, Cdyn, and PaO_2_/FiO_2_. Among them, IOB and POB were considered the primary outcome measures.

**Table 2:** Results of the meta-analysis of outcome measures.

Outcome	Number of studies	Patients PCV/VCV	MD (95% CI)	p Value	Heterogeneity *I*^2^ (%)
Blood loss	4	136/137	−164.07 [-257.60,-70.53]	0.0006	73
IOB (ml)	2	72/72	1.48 [-17.65, 20.62]	0.88	0
POB (ml)	3	118/118	0.25 [-0.02, 0.52]	0.07	0
Hb (g/dl)	3	118/118	0.62 [-0.29, 1.53]	0.18	0
HCT (%)	8	329/329	−2.79 [-3.50, −2.07]	<0.00001	86
Ppeak (cmH_2_O)	4	208/209	0.06 [-0.91, 1.02]	0.91	93
CVP (mmHg)	6	237/238	−0.60 [-1.56, 0.37]	0.23	27
HR (beat/min)	6	237/238	1.91 [-1.25, 5.06]	0.24	86
MAP (mmHg)	4	68/68	6.32 [5.34, 7.31]	<0.00001	0
Cdyn (ml/cmH_2_O)	6	160/161	16.64 [7.98, 25.29]	0.0002	35
PaO^2^/FiO^2^mmHg					

PCV, pressure controlled ventilation; VCV, volume controlled ventilation; MD, mean difference; IOB, intra-operation blood loss; POB, post-operation blood loss; Hb, hemoglobin; HCT, hematocrit; CVP, central venous pressure; HR, heart rates; MAP, mean arterial pressure; Cdyn, dynamic compliance.

#### 3.4.1 Intra-operation blood loss

**Fig. 3:**
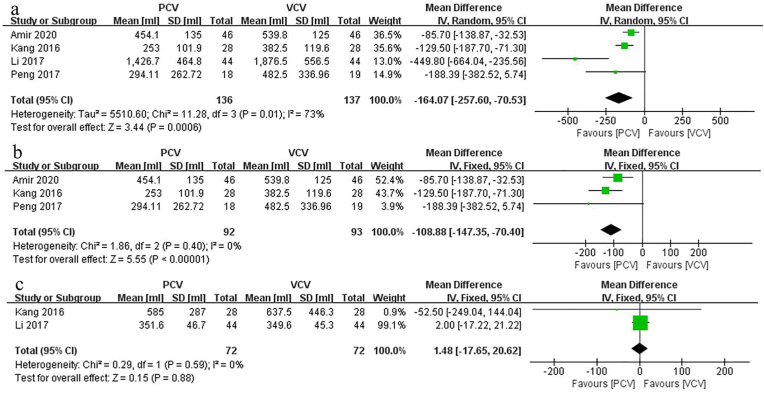
Forest plots and meta-analyses. a: intra-operation blood loss; b: sensitivity analysis of intra-operation blood loss. [95% CI: 95% confidence intervals, df: degrees of freedom, Random: random effects model, Fixed: fixed effects model, IV: inverse variance,]; c. Forest plots of the post-operation blood loss. [95% CI: 95% confidence intervals, df: degrees of freedom, Fixed: fixed effects model, IV: inverse variance,].

Four studies compared IOB between PCV and VCV in spine surgery for patients in prone position. Pooled results indicated that IOB in VCV was significantly higher than that in PCV (MD: 164.07; 95% CI: [0.53, 257.60], p = 0.0006, *I*_*2*_ = 73%) (Fig. 3a). Because there was a significant heterogeneity among the studies, a random-effects model was used to pool the data (*I*_*2*_ > 50%, p < 0.1). To avoid biases, sensitivity analysis was conducted. After removing the study of Li et al. [11], the heterogeneity for IOB decreased significantly (*I*_*2*_ = 0%) (Fig. 3b), which means that this study was the main source of heterogeneity. However, after decreasing heterogeneity, the results remained the same (p < 0.00001).

#### 3.4.2 Post-operation blood loss

Two studies compared POB between PCV and VCV in spine surgery performed with prone position. The results indicated that the differences in PCV and VCV were not statistically significant (MD: 1.48; 95% CI: [−17.65, 20.62], p = 0.88, *I*_*2*_ = 0%) (Fig. 3c).

#### 3.4.3 Hemoglobin at time of extubation

**Fig. 4a:**
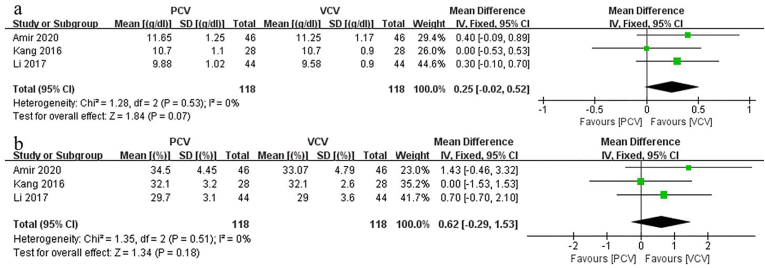
Forest plots of the Hemoglobin. [95% CI: 95% confidence intervals, df: degrees of freedom, Fixed: fixed effects model, IV: inverse variance,]; 4b. Forest plots of the Hematocrit. [95% CI: 95% confidence intervals, df: degrees of freedom, Fixed: fixed effects model, IV: inverse variance,].

Pooling outcomes from the included studies indicated that at the time of extubation, hemoglobin levels between PCV and VCV did not differ significantly (MD: 0.25; 95% CI: [−0.02, 0.52], p = 0.07, *I*_*2*_ = 0%) (Fig. 4a).

#### 3.4.4 Hematocrit at time of extubation

The results of our meta-analysis did not detect a significant difference between PCV and VCV in terms of hematocrit at the time of extubation (MD: 0.62; 95% CI: [−0.29, 1.53], p = 0.18, *I*_*2*_ = 0%) (Fig. 4b).

#### 3.4.5 Ppeak

**Fig. 5a:**
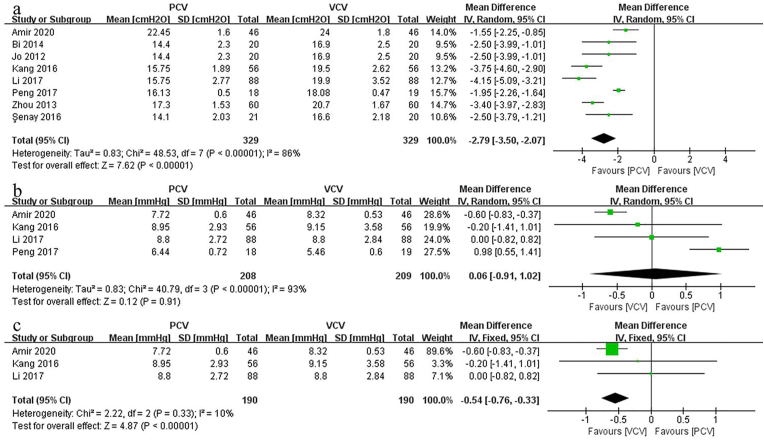
Forest plots of the Ppeak. [95% CI: 95% confidence intervals, df: degrees of freedom, Random: random effects model, IV: inverse variance,]; 5b, c. Forest plots and meta-analyses. b: CVP; c: sensitivity analysis of CVP. [95% CI: 95% confidence intervals, df: degrees of freedom, Random: random effects model, Fixed: fixed effects model, IV: inverse variance,].

A total of 8 studies reported measures of Ppeak. Pooling outcomes indicated a significant difference between PVC and VCV (MD: −2.79; 95% CI: [−3.50, −2.07], p < 0.00001, *I*_*2*_ = 86%) (Fig. 5a). Considering the significant heterogeneity among studies, sensitivity analysis was conducted to further verify the associated factors. However, the above results remained unchanged after sequentially removing each study.

#### 3.4.6 CVP

Four studies compared the CVP between PCV and VCV in prone position spinal surgery. The combined results indicated that CVP was not significantly higher in VCV than in PCV (MD: 0.06; 95% CI: [−0.91, 1.02], p = 0.91, *I*_*2*_ = 93%) (Fig. 5b). Because of the significant heterogeneity among the studies, sensitivity analysis was performed (when *I*_*2*_ > 50%). After removing the study of Peng et al. [17], the heterogeneity for CVP decreased significantly (*I*_*2*_ = 10%), which means that this study was the main source of heterogeneity. Moreover, the results changed after the heterogeneity decreased, which indicates that VCV was significantly and substantially better than PCV in terms of CVP (MD: 0.54; 95% CI: [−0.33, 0.76], p < 0.00001, *I*_*2*_ = 10%) (Fig. 5c).

#### 3.4.7 HR

**Fig. 6a:**
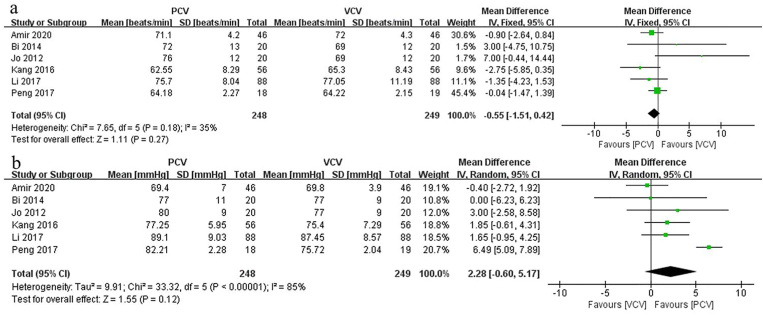
Forest plots of the heart rates. [95% CI: 95% confidence intervals, df: degrees of freedom, Fixed: fixed effects model, IV: inverse variance,]; 6b. Forest plots of the mean arterial pressure. [95% CI: 95% confidence intervals, df: degrees of freedom, Random: random effects model, IV: inverse variance,].

Six studies compared HR between PCV and VCV in spine surgery performed with prone position. Pooled results found that there was no significant difference between the two modes in terms of HR (MD: −0.55; 95% CI: [−1.51, 0.42], p = 0.27, *I*_*2*_ = 35%) (Fig. 6a).

#### 3.4.8 MAP

Six studies compared MAP between PCV and VCV in prone position spinal surgery. Pooled results indicated that there was no significant difference between the two modes in terms of MAP (MD: 2.28; 95% CI: [−0.60, 5.17], p = 0.12, *I*_*2*_ = 85%) (Fig. 6b).

#### 3.4.9 Cdyn

**Fig. 7a:**
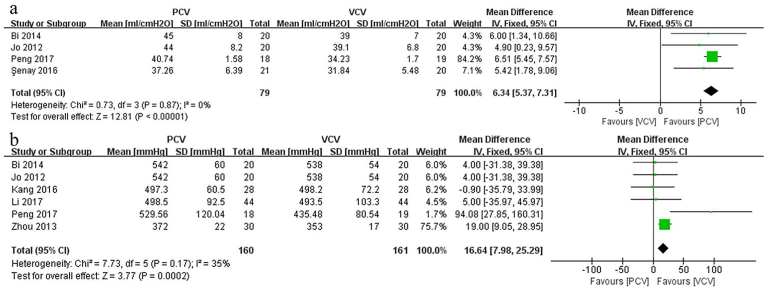
Forest plots of the dynamic compliance. [95% CI: 95% confidence intervals, df: degrees of freedom, Fixed: fixed effects model, IV: inverse variance,]; 7b. Forest plots of the PaO_2_/FiO_2_. [95% CI: 95% confidence intervals, df: degrees of freedom, Fixed: fixed effects model, IV: inverse variance,].

Four studies compared Cdyn between PCV and VCV in spine surgery performed with prone position. Pooled results indicated that Cdyn was significantly greater in PCV than in VCV (MD: 6.34; 95% CI: [5.37, 7.31], p < 0.00001, *I*_*2*_ = 0%) (Fig. 7a).

#### 3.4.10 PaO_2_/FiO_2_

Regarding PaO2/FiO2, 6 studies were enrolled in our meta-analysis. Pooling the results showed that PCV had a higher PaO_2_/FiO_2_ value than VCV (MD: 16.64; 95% CI: [7.98, 25.29], p = 0.0002, *I*_*2*_ = 35%) (Fig. 7b).

### 3.5 Publication bias

Funnel plot asymmetry tests are currently conducted to evaluate publication bias, but such tests are usually only applied when at least 10 studies are included in a meta-analysis. Eight RCTs were enrolled in this meta-analysis, and therefore the power of the funnel plot tests was low. Thus, in our meta-analysis, we did not evaluate publication bias or meta-regression.

## 4 Discussion

When performing spinal surgery in prone position, the traditional mechanical ventilation mode option is VCV because the ventilation airflow in this mode is relatively stable. However, PCV has gradually become of clinical interest in recent years, as studies have shown that it can guarantee the even distribution of gas ventilation to the whole lung field [19]. In addition, increasing clinical attention has been given to the role of ventilation mode in various difficult anesthesia management situations (such as the position of the patient). Most spinal surgeries need to be performed in the prone position, which negatively affects cardiorespiratory fitness [3]. Given the above factors, the anesthesia management of spinal surgery in prone position is more complicated than that of surgery in supine position. Currently, there is no specific evaluation standard in the clinic to test whether PCV or VCV is the optimal regimen for patients undergoing prone position spinal surgery. Therefore, we performed this meta-analysis.

During prone position spinal surgery when combined with mechanical ventilation therapy, blood loss is one of the most common adverse events related to surgical prognosis. In our study, a pooled analysis of 4 studies showed that the amount of intraoperative blood loss in the VCV group was significantly more than that in the PCV group in patients undergoing prone position spinal surgery. Additionally, because of the high heterogeneity (*I*_*2*_ = 73%) for this result, sensitivity analysis was conducted by removing the Li et al. study [11]. Subsequently, the pooled results of the remaining 3 studies indicated *I*_*2*_ = 0, and significant differences in IOB were still present between the PCV group and VCV group. By comparing the demographic and clinical characteristics among the 4 enrolled studies, we found that the largest difference involved the complexity of the lumbar surgery undergone by the patients. Li et al. [11] studied patients with lumbar tuberculosis and tumors, which implied a relatively complicated surgical procedure and long operation time. In contrast, the patients in the other studies were treated by a simple procedure, such as discectomy and fusion. Therefore, the surgical complexity was the primary source of high heterogeneity, and Fig. 3a clearly showed that the amount of hemorrhage in the study of Li et al. [11] was considerably higher than that in the remaining 3 studies. Collectively, after removal of the heterogeneity, the results of our meta-analysis still indicated that IOB for patients in the VCV group was higher than that for patients in the PCV group (Fig. 3b). Contrary to our findings, Lauren et al. [7] drew different conclusions by retrospectively examining patients who underwent elective spine surgery in prone position, which indicated that there was no statistically significant difference in IOB (MD: −163.3; 95% CI: [−580.8 to 254.3], p = 0.44) between PCV and VCV. Several possible reasons may account for the above mentioned inconsistent results. First, the study of Lauren et al. [7] had a retrospective design. Thus, the possibility of bias was relatively large, and the low credibility made the Lauren et al. [7] study unconvincing. Second, ventilation mode is only one of many factors affecting IOB for patients undergoing prone position surgery; the spinal surgery type, duration of the operation, pathology (malignant or benign disease), and proficiency of the surgeon can also affect IOB. As the influence of other factors increases, the effect of ventilation mode on IOB may be offset. In summary, compared to PCV, VCV can result in an increased IOB for patients undergoing prone position spinal surgery. However, limited by the number of enrolled studies and evaluation indicators, the specific mechanism of the relationship between ventilation mode and IOB cannot be further concluded. Further RCTs are needed to characterize the subgroups of surgical procedures that are associated with IOB.

Unlike IOB, POB exhibited no difference between the PCV group and VCV group among patients who underwent spine surgery in the prone position (MD: 1.48; 95% CI: [−17.65, 20.62], p = 0.88, *I*_*2*_ = 0%). This means that the mechanical ventilation mode had no significant effect on postoperative bleeding. Additionally, the results of our meta-analysis found that the Ppeak (MD: 2.79; 95% CI: [2.07, 3.50,], p < 0.00001, *I*_*2*_ = 86%) and CVP (MD: 0.54; 95% CI: [0.33, 0.76], p < 0.00001, *I*_*2*_ = 10%) of the VCV group were significantly higher than those of the PCV group. Therefore, the amount of IOB might be associated with the Ppeak and CVP, which is consistent with previous findings. Koh et al. [20] found that airway pressure could predict intraoperative surgical blood loss during prone position spinal surgery. Malhotra et al. [21] reported that increased mean airway pressure and intra-abdominal pressure may aggravate IOB. These findings highlight the fact that the Ppeak is sufficient to reduce cardiac compliance and increase cardiac preload, contributing to CVP growth. In addition, the valveless epidural vertebral venous system is characterized by a weak vessel wall and lower venous pressure, and an elevated Ppeak and intra-abdominal pressure could easily compress the inferior vena cava and cause blood to flow back into the vertebral venous system during surgery in prone position [22,23].

The reason that the patients in PCV group had a lower Ppeak than in the VCV group was due to specific features of PCV: this ventilation mode uses high initial flow rates in the early stage of inspiration, while the inspiratory flow patterns decelerate until the end of inspiration. In this way, PCV can provide a lower Ppeak and a more even distribution of gas ventilation [24,25]. Likewise, in patients who underwent laparoscopic cholecystectomy, which involves positioning similar to the prone state, Sen et al. [26] and Gupta et al. [27] reported lower Ppeak and CVP levels in the PCV group than in the VCV group. In summary, for patients receiving prone position spinal surgery and ventilating with the same preset tidal volume, the PCV group exhibited lower Ppeak and CVP levels than the VCV group.

Additionally, Hb and HCT at the time of extubation exhibited no significant difference between the two groups, which suggests that intraoperative blood loss is not positively and linearly related to Hb and HCT. This could be due to various factors, such as reflex vasoconstriction and fluid shift [28], which could compensate for the effect of blood loss. Therefore, Hb and HCT are not suitable as evaluation indices to appraise the effect of mechanical ventilation mode on blood loss in patients underwent prone position spinal surgery.

Our pooled results showed that the PCV group displayed significantly higher intraoperative PaO2/FiO2 than the VCV group. However, in the meta-analysis of Jiang et al. [29], different results were obtained. They recruited 3 studies claiming different surgery (laparoscopic prostatectomy, esophagectomy, and lumbar surgery) in prone position, but there was no significant difference identified for PaO^2^/FiO^2^ between PCV group and VCV group among 3 studies (WMD, 1.65; N = 110; p = 0.90; *I*_*2*_ = 0%). Regarding that result in contrast to our study, several reasons might contribute to this: first, the patients of 3 studies have 3 different surgical methods. Despite the heterogeneity of included studies reached zero (*I*_*2*_ = 0%), 3 studies vary wildly in terms of the operation time, surgical procedure, and cardiorespiratory function levels, which will influence the effect of gas ventilation and exchange. Second, anesthetic regimen had no unified standard among 3 studies. For instance, FiO^2^ is an important parameter affecting oxygenation. Besides, one of 3 studies used one-lung ventilation in prone, which would make human-caused intrapulmonary shunt to decrease PaO^2^. In the end, alveolar-arterial oxygen difference and anatomical dead-space display important implications for oxygenation, and our studies amounts are not sufficient to perform the subgroup analysis for these two factors. More RCT studies are needed to research the effect of alveolar-arterial oxygen difference and anatomical dead-space on mechanical ventilation.

According to this meta-analysis, the PCV group presented a higher Cdyn value than the VCV group. This is mainly because PCV has a lower Ppeak than VCV [Cdyn = Tidal volume/(Ppeak - positive end inspiratory pressure)] [30]. Our pooled data indicated that although the prone position decreased the Cdyn of patients, PCV maintained a better Cdyn than VCV during spine surgery. However, it is important to note that the patients of all included studies had a BMI less than 30 kg/m2. Considering that the preset airway pressure in PCV grows approximately and exponentially with the decrease of Cdyn, future high-quality RCTs should research the effect of mechanical ventilation on obese patients undergoing prone position spinal surgery.

In terms of hemodynamics, this meta-analysis adopted 2 related parameters: HR and MAP, but neither demonstrated significant differences between PCV and VCV. Similarly, these results were consistent with previous studies. Messeha et al. [31]. and Jaju et al. [32]. evaluated the effect of PCV versus VCV on hemodynamic parameters and found hemodynamic parameters were comparable between two groups. In contrast, changing from the supine to the prone position affects hemodynamic variables, as manifested by a significant decline in HR and BP values. Channabasappa et al. [33] found that during lumbar surgery, the HR and MAP of patients were significantly higher in supine position than in prone position. Al-Dessoukey et al. [34] claimed that MAP decreased by 14 mmHg upon changing patients from the supine to the prone position during surgery. From the above results, we conclude that mechanical ventilation has a minimal impact on the HR and MAP of patients in the prone position during spine surgery.

This meta-analysis was designed to retrieve all currently available RCTs; however, there were some limitations in this review. First, there were some studies that did not provide detailed information about allocation concealment or blinding of outcome assessments, which in turn might have contributed to the risk of bias. Second, the number of enrolled studies reporting IOB was small, and some relative subgroup analyses could not be successfully performed. Third, the accurate recording times of several parameters were not uniform. Fourth, most articles did not provide patient disease definitions, specificity of the surgical procedure, or postoperative complications. Therefore, considering these heterogeneities, we need to interpret our results cautiously.

## 5 Conclusion

This meta-analysis suggests that compared with the VCV mode with the same preset tidal volume, the PCV mode yielded less IOB, a lower P-peak and CVP, and a higher PaO2/FiO2 value for patients undergoing prone position spinal surgery. However, it exhibited no significant difference between PCV and VCV in terms of hemodynamic variables (HR and MAP). These findings suggest that PCV might be a viable option to mechanical ventilation for patients undergoing lumbar spine surgery in the prone position. This means that PCV would reduce the operation time and total blood loss of patients with spine surgery and benefit to early rehabilitation promoting recovery. However, cautions should be applied since these recommendations are based only on a varied group of patients. In future studies, researchers also need to compare different ventilation modes in terms of anatomical dead space and left ventricular function. More importantly, we need to follow up the patients after spine surgery to study whether the choice of intraoperative ventilation strategy will have a long-term impact on the patient's prognosis. Therefore, we anticipate confirmation of this conclusion through further well-designed RCTs with larger samples of patients.

## Ethical approval

Not needed.

## Sources of funding

This study was supported by grants from 10.13039/501100001809National Natural Science Foundation of China (No. 31971275).

## Author contribution

H.J., H.YX. and L.SM. wrote the main manuscript text, H.ZX. and L.WZ prepared tables and figures. All authors reviewed the manuscript.

## Registration of research studies

1. Name of the registry:

PROSPERO.

2. Unique Identifying number or registration ID:

CRD42020196916.

3. Hyperlink to your specific registration (must be publicly accessible and will be checked):

https://www.crd.york.ac.uk/prospero/display_record.php?RecordID=196916.

## Guarantor

Hong Wang.

## Consent

No.

## Consent for publication

Not applicable.

## Provenance and peer review

Not commissioned, externally peer-reviewed.

## Declaration of competing interest

The authors declare no relevant conflict of interest.
